# Identification and functional characterization of m1A-related genes in colorectal cancer: implications for prognosis, immune infiltration, and therapeutic strategies

**DOI:** 10.3389/fonc.2025.1532602

**Published:** 2025-02-18

**Authors:** Lan Sun, Liwei Huangfu, Fang Li, Yuhui Yan, Ruiping Kong, Kun Ji, Jiachun Li

**Affiliations:** ^1^ Department of Pharmaceutical Engineering, Jiangsu Food and Pharmaceutical Science College, Huaian, Jiangsu, China; ^2^ State Key Laboratory of New-tech For Chinese Medicine Pharmaceutic Process, Jiangsu Kanion Pharmaceutical Co., Ltd., Lianyungang, Jiangsu, China; ^3^ Department of Grain and Food Pharmacy, Jiangsu Vocational College of Finance and Economics, Huaian, Jiangsu, China

**Keywords:** colorectal cancer (CRC), m1A-related genes, tumor microenvironment (TME), prognostic model, immune infiltration

## Abstract

**Introduction:**

Colorectal cancer (CRC), characterized by its complex genetic heterogeneity and varied responses to treatment, is a leading cause of cancer-related mortality worldwide. The role of N1-methyladenosine (m1A)-related genes in tumor biology remains underexplored. This study aimed to investigate the prognostic value of m1A-related genes in CRC, characterize their role in tumor molecular subtyping, and explore their influence on the tumor microenvironment (TME) and immune infiltration.

**Methods:**

To identify prognostic markers, univariate Cox analysis was performed using multiple datasets, including TCGA and GEO, identifying 43 m1A-related genes. Four distinct molecular subtypes of CRC were defined based on the expression of these genes using non-negative matrix factorization (NMF). Immune infiltration analysis was conducted, and the TIDE algorithm was used to predict response to immune checkpoint inhibitors (ICIs). Furthermore, a prognostic model based on m1A-related genes was constructed and validated across multiple datasets.

**Results:**

The results demonstrated that the four CRC molecular subtypes exhibited significant differences in survival outcomes and clinical characteristics. Stromal cells showed higher m1A scores, suggesting a regulatory role in the TME. There was a positive correlation between m1A-related gene expression and immune checkpoint genes. Moreover, the constructed prognostic model showed robust predictive performance and outperformed other recently published models.

**Discussion:**

The findings suggest that m1A-related genes are not only valuable biomarkers for CRC prognosis but also have significant implications for the immune landscape and could serve as potential targets for therapeutic intervention, particularly in the context of immunotherapy. For instance, SLC12A2 was found to enhance invasion, proliferation, and migration of colorectal cancer cells while inhibiting apoptosis. Further studies are needed to understand the functional roles of m1A modifications across different cell types within the TME.

## 1 Introduction

Colorectal cancer (CRC), one of the most common cancers globally that contributes to approximately 10% of all cancer cases and deaths worldwide, remains a leading cause of cancer-related mortality (1, 2). Despite advances in prevention, early diagnosis, and treatment, CRC continues to pose a significant public health challenge, particularly in low- and middle-income countries where the incidence and mortality rates are on the rise (3–5). The multifactorial etiology of CRC, which involves genetic, epigenetic, environmental, and lifestyle factors, contributes to its complex pathophysiology (6, 7). Accordingly, there is an urgent need to better understand the underlying mechanisms driving CRC development, progression, and therapeutic resistance to improve patient outcomes.

A defining feature of CRC is tumor heterogeneity that significantly and adversely affects prognosis and therapeutic responses (8, 9). CRC tumors can substantially vary in their molecular and genetic characteristics, which results in considerable differences in growth patterns, metastatic potential, and sensitivity to treatments (10, 11). This heterogeneity can manifest both on the inter-patient level, where different patients present with tumors with distinct genetic and molecular profiles, and on the intra-patient level, where different regions of the same tumor exhibit varying features (12–14). Consequently, identifying robust molecular subtypes reflecting the biological diversity of CRC can assist in precise risk stratification and personalized treatment planning (15). To date, numerous studies, including those from The Cancer Genome Atlas (TCGA), have identified molecular subtypes of CRC based on key genetic and transcriptomic alterations, such as mutations in KRAS, BRAF, and TP53, as well as microsatellite instability (MSI) status (16–18). However, to uncover additional layers of complexity in CRC, a more thorough exploration into other regulatory mechanisms, such as RNA modifications, is still warranted.

Recently, RNA modifications have emerged as important regulatory layers capable of modulating gene expression and implicated in numerous physiological and pathological processes, including cancer (19). Among over 170 known chemical modifications of RNA, N1-methyladenosine (m1A) and N6-methyladenosine (m6A) are prominent in eukaryotic cells (20, 21). Extensive research on m6A demonstrated that m6A can regulate RNA splicing, translation, and stability, all of which affects various aspects of cancer biology (22, 23). In its turn, m1A is a relatively less explored RNA modification, and its role in cancer, including CRC, remained poorly understood. m1A modifications can alter RNA structure and affect ribosomal RNA (rRNA) and transfer RNA (tRNA) function, thereby impacting the protein synthesis machinery, which is frequently dysregulated in cancers (24, 25). Recent research also suggested that m1A-related genes might be involved in oncogenic pathways and could serve as potential biomarkers for cancer prognosis. Yet, their specific role in CRC remains unclear.

The tumor microenvironment (TME) plays a crucial role in CRC progression and therapeutic resistance. The TME in CRC consists of various cell types, including immune cells, fibroblasts, endothelial cells, and extracellular matrix components, all of which interact with cancer cells in complex ways to either promote or inhibit tumor growth (26, 27). There is also evidence to suggest that immune landscape of CRC, characterized by immune cell infiltration such as tumor-associated macrophages (TAMs), T cells, and myeloid-derived suppressor cells (MDSCs), can significantly impact the effectiveness of treatments such as chemotherapy, targeted therapy, and immunotherapy (28). While the interplay between tumor cells and the immune system was reported to be affected by several factors, including genetic and epigenetic modifications, the relationship between RNA modifications like m1A and immune modulation in CRC is not yet fully understood. Therefore, a thorough investigation into how m1A-related genes influence immune infiltration and TME composition can provide new insights into immunotherapeutic strategies for CRC.

Recent advances in high-throughput sequencing technologies, such as single-cell RNA sequencing (scRNA-seq) and spatial transcriptomics, have enabled researchers to dissect the cellular composition and spatial organization of tumors at an unprecedented resolution (29). Such technologies are particularly valuable in studying CRC, where of disease progression and patient prognosis are largely determined by both tumor heterogeneity and the TME. scRNA-seq allows for identifying distinct cell populations within the tumor and the TME, revealing heterogeneity that would be masked in bulk RNA analyses (30). Spatial transcriptomics complements this by providing spatial context, which is essential for understanding cell-cell interactions and the functional organization of the TME. These technologies can provide comprehensive insights into how m1A-related genes contribute to the heterogeneity of CRC and affect cellular communication within the tumor.

In the present study, aiming to identify novel biomarkers that could help stratify patients based on risk and predict therapeutic outcomes, we explored the prognostic value of m1A-related genes in CRC. Our hypothesis was that m1A-related genes are critically involved in the regulation of CRC tumor behavior, affecting processes such as tumor cell proliferation, metastasis, and immune modulation. By characterizing the expression patterns of m1A-related genes in CRC and defining molecular subtypes based on their expression, we sought to provide novel insights into the molecular mechanisms underlying CRC heterogeneity and identify potential targets for therapeutic intervention. Overall, the results of the present study provide a comprehensive analysis of m1A-related genes in CRC, highlighting their potential as prognostic biomarkers and therapeutic targets.

## 2 Methods

### 2.1 Acquisition and processing of transcriptomic data

The RNA expression profiles (*n* = 606) and the corresponding clinical data of colorectal cancer from the TCGA database were selected as the training group, which was then used to construct the model. The validation group was used to test the stability and accuracy of the model. All data were converted to TPM format and log2-transformed for subsequent analysis. In addition, microarray datasets from the GEO database, including GSE12945 (*n* = 62), GSE17536 (*n* = 177), GSE17537 (*n* = 55), GSE38832 (n = 122), GSE39582 (*n* = 579), GSE41258 (*n* = 182), GSE87211 (*n* = 196), and GSE103479 (*n* = 155), were used as the validation group. Furthermore, colorectal cancer microarray data from GSE110224 (T = 17, *n* = 17), GSE22598 (T = 17, *n* = 17), and GSE41328 (T = 10, *n* = 10) were included for differential gene analysis. Data correction for the microarray data was performed using the normalizeBetweenArrays function from the limma package.

### 2.2 Acquisition and processing of single-cell and spatial transcriptomic data

The single-cell dataset was obtained from the GEO database for a total of 15 tumor samples: GSE166555 with 13 CRC tumor samples and GSE221575 with 2 CRC tumor samples. R software (version 4.1.3) and the Seurat package were used for data analysis. For cell quality control, mitochondrial content was required to be below 20%, and the range for UMI counts and gene counts per cell was set between 200-20,000 and 200-5,000, respectively. Data normalization, selection of highly variable genes (2,000 genes), and data transformation [eliminating cell cycle effects using the parameter vars.to.regress = c(“S.Score”, “G2M.Score”)] were performed using the functions NormalizeData, FindVariableFeatures, and ScaleData from Seurat. Dimensionality reduction was conducted using UMAP and t-SNE, while clustering was performed using the Louvain algorithm (all from Seurat). To identify differentially expressed genes between clusters or cell types, we used the FindAllMarkers function, with parameters set to *p*-value < 0.05, log2FC > 0.25, and expression proportion > 0.1.

Spatial transcriptomic data were obtained from scCRLM (http://www.cancerdiversity.asia/scCRLM/), including 4 tumor samples. The downstream results, quality-controlled using SpaceRanger software, were analyzed using SCTtransform for data normalization, selection of highly variable genes, and data transformation. The average number of spots amounted to 3,849, with average UMI counts, gene counts, and mitochondrial content of 12,138.6, 3,236.1, and 6%, respectively. Data analysis and visualization were performed using Seurat. Deconvolution analysis was conducted using the conditional autoregression-based deconvolution (CARD) algorithm, with cell type predictions for each spot based on single-cell annotations. Visualization of cell types in the spatial transcriptomic data was performed using the CARD software. The AUCell package was used to calculate signature scores.

### 2.3 Cell annotation analysis

We initially used the following specific markers to annotate different cell types: epithelial cell markers (“EPCAM”, “KRT18”, “KRT19”, “CDH1”); fibroblast markers (“DCN”, “THY1”, “COL1A1”, “COL1A2”); endothelial cell markers (“PECAM1”, “CLDN5”, “FLT1”, “RAMP2”); T cell markers (“CD3D”, “CD3E”, “CD3G”, “TRAC”); NK cell markers (“NKG7”, “GNLY”, “NCAM1”, “KLRD1”); B cell markers (“CD79A”, “IGHM”, “IGHG3”, “IGHA2”); mast cell markers (“KIT”, “MS4A2”, “GATA2”). Based on this annotation, we isolated and clustered epithelial cells for the subsequent analysis to explore tumor heterogeneity, and generated visualizations including UMAP, t-SNE, bar plots, and heatmaps.

### 2.4 Acquisition of m1A-related genes and related genes

From the literature, we obtained 10 m1A-related genes (TRMT10C, TRMT61B, TRMT6, TRMT61A, ALKBH1, ALKBH3, YTHDF1, YTHDF2, YTHDF3, and YTHDC1). We then identified additional genes that significantly correlated with these 10 genes and thus also had prognostic value.

### 2.5 Acquisition of prognostic genes and consensus clustering analysis

Correlation analysis was performed between 10 m1A-related genes and 43 prognostic genes (which were related to prognosis in at least 5 datasets), identifying 43 genes associated with m1A. We then used three datasets with tumor and adjacent normal samples, along with the TCGA dataset, to determine differentially expressed genes between tumor and normal tissues. This resulted in the identification of 35 m1A-related genes (PCOLCE2, PEG3, SCG2, FABP4, RBM47, AOC3, CRYAB, KLK6, STIL, CALB2, SLC12A2, TAGLN, FLNA, MAP1B, RAB3B, INHBB, CD3G, AKT3, HLX, GDI1, PLAT, ABLIM3, MRS2, ACVR1, BEAN1, NPR3, TAPBPL, CAV2, APOL6, HOXC6, TNIK, GUCY2C, PLK2, PTPN14, and TMEM204). Using these 35 prognostic genes, we then applied a clustering discovery method called nonnegative matrix factorization (NMF) on the TCGA-CRC cohort, using the NMF package. The optimal number of clusters was determined using the cophenetic metric.

### 2.6 SNV analysis

Single nucleotide variant (SNV) mutation data were downloaded from the TCGA database. The maftools package were used to evaluate the tumor mutation burden (TMB) of each sample. Differences between risk groups were analyzed using the Wilcoxon test, with a *p*-value < 0.05 considered to indicate statistical significance.

### 2.7 Analysis of cell-cell communication

The CellChat package was used to evaluate potential intercellular communication. We also used the CellChat function to import the normalized gene expression matrix to create the CellChat object. The data were then preprocessed using the functions identifyOverExpressedGenes, identifyOverExpressedInteraction, and ProjectData with default parameters. The computeCommunProb, filterCommunication, and computeCommunProbPathway functions were then used to determine any potential ligand-receptor interactions. Finally, the aggregateNet function was employed to generate the cell communication network.

### 2.8 Differential gene analysis and enrichment analysis

To investigate gene expression differences between tumor and adjacent normal samples, we conducted differential gene analysis for GEO and TCGA datasets using the limma package. The genes with adjusted *p*-value (*p*_adj_) < 0.05 and |Fold Change| > 1.2 were considered differentially expressed. The clusterProfiler package was used for enrichment analysis of upregulated and downregulated genes using GSEA, with functional databases HALLMARK, GOBP, and KEGG, and signatures obtained from the msigdb database. Enrichment results were visualized using the enrichplot package.

### 2.9 Establishment of tumor-related risk features

A prognostic model was established using 101 machine learning methods, providing a risk score for each patient based on the algorithm. We used the surv_cutpoint function to determine the cutoff value for grouping, with patients in the TCGA cohort and other cohorts divided into high-risk and low-risk groups. We then evaluated the predictive consistency between the two groups and evaluated accuracy of the model.

### 2.10 Risk features generated by an ensemble machine learning method

To develop a model with high accuracy and stability, we integrated 10 machine learning algorithms and 101 algorithm combinations. The comprehensive algorithms included Random Survival Forest (RSF), Elastic Net (Enet), Lasso, Ridge, Stepwise Cox, CoxBoost, Cox Partial Least Squares Regression (plsRcox), Supervised Principal Components (SuperPC), Generalized Boosted Regression Model (GBM), and Survival Support Vector Machine (survival-SVM). The signature generation procedure unfolded in the following four steps:

1. Univariate Cox regression analysis was used to identify prognostic genes (as described in the previous step) across 9 datasets, including TCGA-CRC;
2. Subsequently, 101 algorithm combinations were applied to fit a prediction model for the TCGA-CRC cohort based on the leave-one-out cross-validation (LOOCV) framework;
3. All models were tested in 8 validation datasets (GEO datasets);
4. For each model, the Harrell’s concordance index (C-index) was calculated across all validation datasets, and the model with the highest average C-index was considered the best.

### 2.11 Prediction of immunotherapy response, IPS analysis, and immune checkpoint analysis

We conducted immunotherapy response prediction using datasets GSE100797 (Melanoma), phs000452 (Melanoma), PRJEB23709 (Melanoma), and GSE35640 (Melanoma). The risk scores for each dataset were calculated to predict immunotherapy response. We also used the TIDE online analysis tool (http://tide.dfci.harvard.edu/) to predict immune response and scores for the TCGA dataset. The IOBR package was used to calculate relevant IPS information, and the differences in IPS between risk groups were evaluated. Finally, we computed the correlations between the expression levels of immune checkpoint genes (“HAVCR1”, “CD28”, “ICOS”, “TNFRSF9”, “IL2RB”, “CD27”, “TNFSF14”, “CD40”, “TNFSF18”, “TNFRSF4”, “TNFRSF18”, “CD276”, “PVR”, “VTCN1”, “CD200”, “C10orf54”, “CD200R1”, “BTLA”, “IDO1”, “TIGIT”, “LAG3”, “CD80”, “CD86”, “LAIR1”, “ADORA2A”, “CTLA4”, “KIR3DL1”, and “CEACAM1”) and risk scores.

### 2.12 Tumor immune infiltration analysis

Using the IOBR package, we determined immune infiltration levels in CRC patients from the TCGA database. To this end, four different evaluation methods, including CIBERSORT, TIMER, MCPcounter, and ESTIMATE, were used. Relative proportions of immune cell infiltration in the TME were quantified and displayed using heatmaps. The results of the ESTIMATE algorithm were used to evaluated the relative abundance of stromal, immune, and tumor cells, and these values were compared across different risk groups.

### 2.13 Drug sensitivity analysis

The R package “oncoPredict” was used to calculate the half-maximal inhibitory concentration (IC50) of commonly used chemotherapy drugs, enabling the subsequent assessment of the relationship between risk scores and drug sensitivity. The Wilcoxon rank-sum test was used to compare IC50 values between the two risk groups.

### 2.14 Cell culture and transfection

This study used human colon epithelial cells (HCoEpiC) and colorectal cancer cell lines (SW480, SW620, LOVO, HCT15). The aforementioned cell lines were sourced from the Cell Bank of the Chinese Academy of Sciences. The culture conditions were as follows: HCoEpiC cells were maintained in the DMEM medium (HyClone, USA) enriched with 10% fetal bovine serum (FBS, BI, Israel) and 1% Penicillin-Streptomycin-Amphotericin B Solution; SW480 and SW620 cells were cultured in the DMEM/F12 medium (Gibco, Thermo Fisher Scientific, USA) supplemented with 10% FBS, 1% L-glutamine (Gibco, Thermo Fisher Scientific, USA), and 1% Penicillin-Streptomycin-Amphotericin B Solution (Gibco, USA); the LOVO cell line was sustained in ATCC-formulated Ham’s F12K medium with 10% FBS, 100 U/mL penicillin, and 100 μg/mL streptomycin; finally, HCT15 cells were grown in DMEM with 10% FBS, 100 U/mL penicillin, and 250 ng/mL streptomycin. All cell lines were maintained in a humidified incubator at 37°C with 5% CO2 to ensure logarithmic growth.

We then conducted transfection experiments on SW620 and LOVO cell lines. To this end, a biotechnology company was commissioned to design and produce a specific shRNA sequence to knock down the expression of SLC12A2 in both cell lines, which yielded SW620 sh-SLC12A2 and LOVO sh-SLC12A2 cell lines. Initially, the two cell lines were detached from culture flasks using a gentle trypsinization process, followed by resuspension in complete medium. This medium contained essential nutrients, vitamins, amino acids, and growth factors to support cell viability and proliferation. Resuspension ensured an even distribution of cells, which was vital for accurate experimental results and consistent growth in subsequent assays. In each well of a 6-well plate, a total of 1 × 10^4^ cells were uniformly distributed, and complete medium was added to reach the final volume of 2 mL per well. After the cells adhered to the plate, we mixed the shRNA and the transfection reagent PolyFast (Catalog No. HY-K1014, MCE, USA) following the manufacturer’s instructions and allowed the mixture to sit at 23°C for 15 min. The transfection mixture was then carefully introduced into the designated wells using a pipette. After a 6-h incubation, the medium was changed with fresh complete medium to optimize nutrient supply. Subsequent experiments were carried out 48 h post-transfection to allow sufficient time for expression of the transfected material and stabilization of cellular conditions for analysis.

### 2.15 Total RNA extraction and RT-qPCR

RT-qPCR was used to evaluate variations in mRNA expression of the SLC12A2 among the groups. To this end, the cells were initially detached from 6-well plates using trypsin (HyClone, USA) and subsequently washed thrice with PBS. The samples were then centrifuged at low temperatures to eliminate supernatant. Subsequently, the cells were lysed by adding an appropriate volume of Trizol (Takara, Japan) following the manufacturer’s instructions. After a 5-min incubation on ice, we sequentially added 200 μL of the chloroform (SINOPHARM, China), along with an equal volume of anhydrous ethanol (SINOPHARM, China) and isopropanol (SINOPHARM, China). Prior to each addition, the solutions were thoroughly mixed and centrifuged at 4°C for 15 min. All organic solvents were removed, and the RNA pellet was left to air dry for 20 min.

Next, we resuspended RNA pellet in 20μL of DEPC-treated water and measured the concentration using a Nanodrop 2000 (Thermo, USA). Following the manufacturer’s guidelines, reverse transcription was performed using the PrimeScript RT Reagent Kit (TaKaRa, Japan) to synthesize cDNA. The resulting cDNA samples were pre-mixed with SYBR GreenER Supermix (TaKaRa, Japan). Real-time quantitative PCR analysis was then performed using a 7500 Real-Time PCR System (Thermo Fisher Scientific, USA), with reaction conditions set according to the SYBR GreenER Supermix protocol. Relative expression levels of SLC12A2 were evaluated using the 2^–ΔΔCt^ method, normalizing to the β-actin as the reference gene.

### 2.16 CCK8 assay

At 48 h post-transfection, the cells were detached using trypsin (KeyGEN, China) and evenly dispersed in the complete medium. Based on the cell count, the cells were plated in a 96-well plate at a density of 5000 cells per well. To ensure accuracy, three replicates were set up for each group. On observation of cell adherence under a microscope, we mixed the CCK8 reagent (KeyGEN, China) with complete medium to reach a total volume of 200 μL per well. The mixture was then quickly added to the wells covered with aluminum foil to protect from the light. Absorbance at 450 nm was measured after 1.5 h using a suitable spectrophotometer. This measurement facilitated the assessment of cell viability or proliferation through the intensity of color generated by CCK8 reagent. This procedure was then repeated at 24, 48, and 72h to evaluate cell viability over time.

### 2.17 Flow cytometry for apoptosis

For apoptosis analysis, SW620 sh-NC and SW620 sh-SLC12A2 cells were harvested by centrifugation at 2,000 rpm for 5 min, facilitating the cells’ pelleting for subsequent analysis. Adherent cells were detached using trypsin without EDTA, followed by two washes with PBS and another centrifugation (2,000 rpm for 5 minutes) to collect 1–5 × 10^5^ cells. The cells were resuspended in 500 μL of the binding buffer. After thoroughly mixing in 5 μL of Annexin V-FITC, 5 μL of propidium iodide were added, and the entire solution was gently mixed. The samples were incubated in the dark at room temperature for 5 to 15 min. Finally, the samples were analyzed using a flow cytometer within 1h.

### 2.18 Transwell assay

In the course of the study, a layer of Matrigel (Thermo, USA) was applied to the inner surface of the chambers, diluted at a 1:9 ratio, with 30 μL deposited in each chamber, and allowed to dry. Subsequently, 600 μL of complete medium were added to each well of a 24-well plate. At 48h post transfection, the cells were detached and resuspended in FBS-free medium. To ensure experimental precision, the cell suspension was modified to achieve a concentration of 30,000 cells per well, with 200 μL added to each chamber. The chambers were then incubated for 24 h. Following this incubation period, the medium in chambers was discarded, and non-invading cells were removed using a moist cotton swab. To further analyze results, the chambers were fixed with 4% paraformaldehyde for 20 min. After three washes with PBS, a 0.1% crystal violet staining solution was added and left for 20 min. Chambers were washed again with PBS and allowed to dry. Finally, images were captured under a microscope for further analysis and discussion of the experimental results.

### 2.19 Protein extraction and western blot analysis

Western blot analysis was performed to evaluate the differences in protein expression levels of cleaved Bcl-2, caspase-3, vimentin, and E-cadherin between the SW620 sh-NC and SW620 sh-SLC12A2 cell lines. Initially, the cells were detached from the culture plates. A lysis buffer was prepared by combining RIPA buffer (Beyotime, China) with protease inhibitors (Beyotime, China) at a ratio of 100:1. This mixture was added to the centrifuge tubes containing the cell pellet, which was thoroughly resuspended. Subsequently, the cells were lysed using an ultrasonic homogenizer with parameters set to 40% amplitude, applying 1 sec pulses for three cycles. The lysate was then kept on ice for 30 min, with intermittent mixing and centrifugation per 10 min. Following the preparation of the lysis buffer, the lysate was centrifuged at 10,000 RPM for 15 min at low temperatures. The supernatant was carefully collected, and the protein concentration was determined. Based on this measurement, an appropriate volume of sample buffer was added to each sample. The samples were then heated in a metal bath at 95°C or about 5 min to denature the proteins and subsequently allowed to cool.

For protein separation, we used 10%SDS-PAGE (20µg/lane) and conducted electrophoresis at 100 V, followed by transfer to a 0.45 µM PVDF membrane (GE Healthcare, USA). The membrane was blocked with a rapid blocking solution (Beyotime, China) for 10 min, followed by three washes with TBST containing 0.1% Tween-20. It was then incubated overnight at 4°C with the corresponding primary antibody. After 16 h, the membrane was washed thrice with TBST and incubated at room temperature for 1.5 h with an HRP-conjugated secondary antibody. Protein bands were visualized using enhanced chemiluminescence (ECL, Beyotime, China). The antibodies used in this study were sourced from Proteintech Group, Inc.

### 2.20 Statistical analyses

All data processing, statistical analyses, and visualizations were performed using R software (version 4.1.3). The correlation between two continuous variables was assessed using Pearson or Spearman correlation coefficients. The chi-square test was used for comparing categorical variables, while the Wilcoxon rank-sum test or t-test was used for comparing continuous variables. Cox regression and Kaplan-Meier analysis were performed using the survival package.

## 3 Results

### 3.1 Characterization of the target gene set

**Figure 1:**
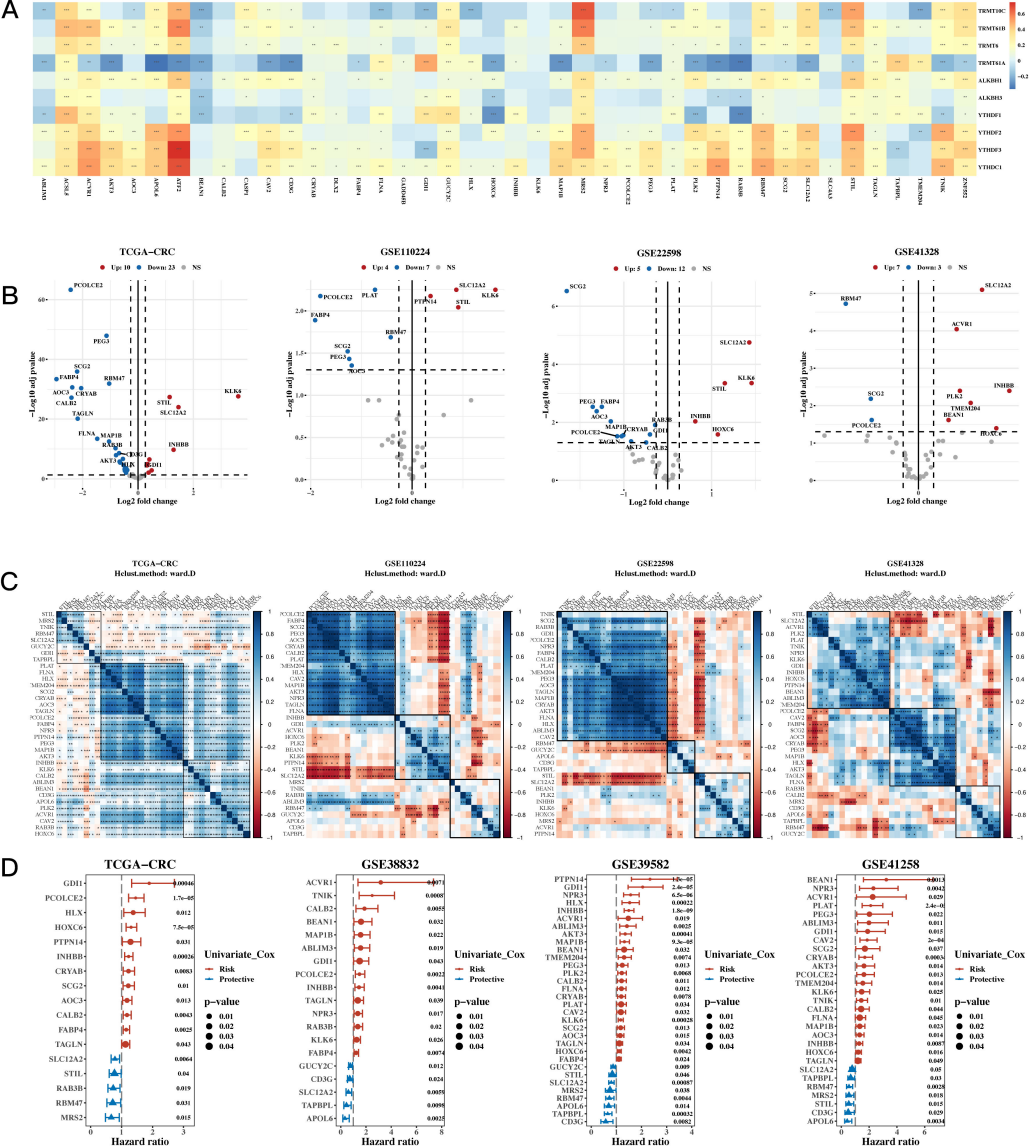
Characterization Results of the Target Gene Set. **(A)** Relationship between 10 m1A-related genes and 43 mitochondrial-related gene sets (correlation heatmap). A p value < 0.05 was considered statistically significant (* p < 0.05; ** p < 0.01; *** p < 0.001). **(B)** Differential expression of m1A-related genes between tumor and adjacent normal tissues across TCGA, GSE110224, GSE22598, and GSE41328 (volcano plots). **(C)** Correlation heatmap of differentially expressed genes from TCGA, GSE110224, GSE22598, and GSE41328 datasets. **(D)** TCGA and three GEO validation datasets (hazard ratio (HR) forest plot).

To analyze the data, univariate Cox analysis was first conducted using TCGA along with eight GEO validation datasets; the results were plotted using the forest plot (see **Figure 1D**). The genes that were prognostic in at least five datasets were selected, resulting in a total of 43 prognostic genes. The correlation between 10 m1A-related genes and 43 prognostic genes is shown in **Figure 1A**, highlighting significant associations and identifying m1A-related genes linked to these prognostic markers. Next, differential expression analysis was performed on TCGA, GSE110224, GSE22598, and GSE41328 between tumor and adjacent normal tissues (see **Figure 1B**), and a total of 35 differentially expressed genes were identified in at least one dataset. **Figure 1C** shows a heatmap of the correlation of the expression of these differentially expressed genes for TCGA, GSE110224, GSE22598, and GSE41328, illustrating the strength and direction of the correlations across datasets. In **Figure 1C**, the color gradient represents the correlation coefficients, with red indicating positive correlations and blue indicating negative correlations; significant correlations are marked with asterisks. This visualization highlights clusters of genes with similar expression patterns, which may reflect shared biological functions or regulatory mechanisms.

### 3.2 Functional characterization - molecular subtyping

**Figure 2:**
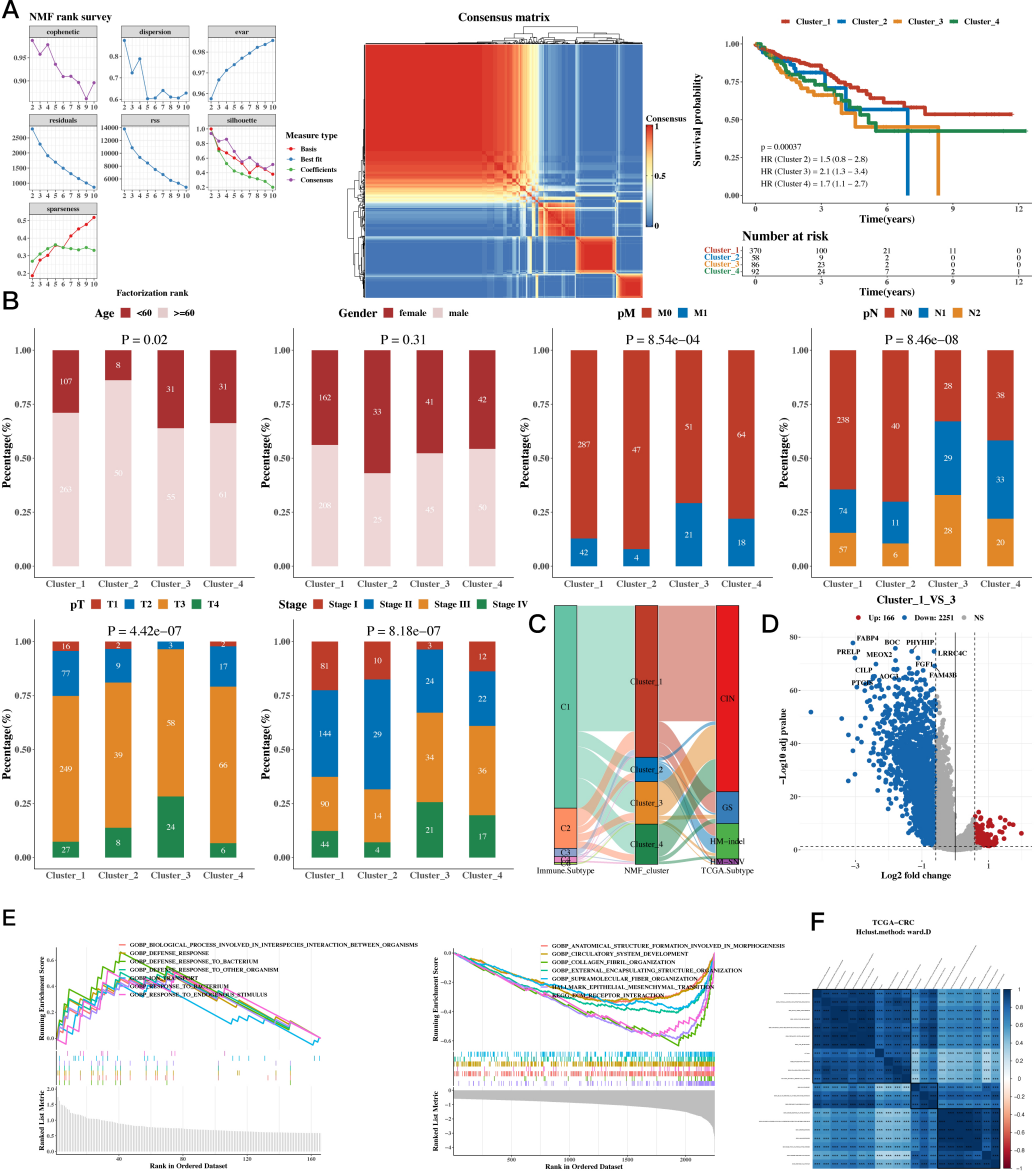
Functional Characterization - Molecular Subtyping Results. **(A)** NMF clustering results, consensus heatmap, and survival analysis for 35 prognostic genes. **(B)** Association between NMF classification and clinical indicators such as age, gender, stage, and pathological grade (bar plots). **(C)** Composition of TCGA immune subtypes and TCGA subtypes as compared to NMF groups (Sankey diagram). **(D)** Differential gene expression between C1 and C3 subtypes (volcano plots). **(E)** Upregulated and downregulated genes between C1 and C3 (GSEA). **(F)** Enriched pathways and ssGSEA scores of the 35 m1A-related genes (correlation heatmap).

The NMF algorithm was used to perform consensus clustering of the 35 prognostic genes. Based on the clustering results, four groups were found to be the most suitable, and the consistency clustering heatmap and survival analysis results for the four groups were presented. The results indicated a significant difference in survival between C1 and C3, with C1 having a better prognosis (see **Figure 2A**). The subsequent analysis on whether the composition of clinical indicators such as age, gender, stage, and pathological grade differed among the four groups yielded significant differences (**Figure 2B**). We then compared the immune subtypes and TCGA subtypes of TCGA with the NMF groups (**Figure 2C**). Since there was a significant survival difference between C1 and C3, differential gene analysis was performed between C1 and C3 (see **Figure 2D**). Enrichment analysis was conducted for upregulated and downregulated genes, focusing on the respective functions of C1 and C3 (see **Figure 2E**). We then calculated the correlation between enriched pathways and the ssGSEA scores of the 35 m1A genes, which was followed by a heatmap analysis of the correlation (**Figure 2F**).

### 3.3 Functional characterization - single-cell and spatial transcriptomics

**Figure 3:**
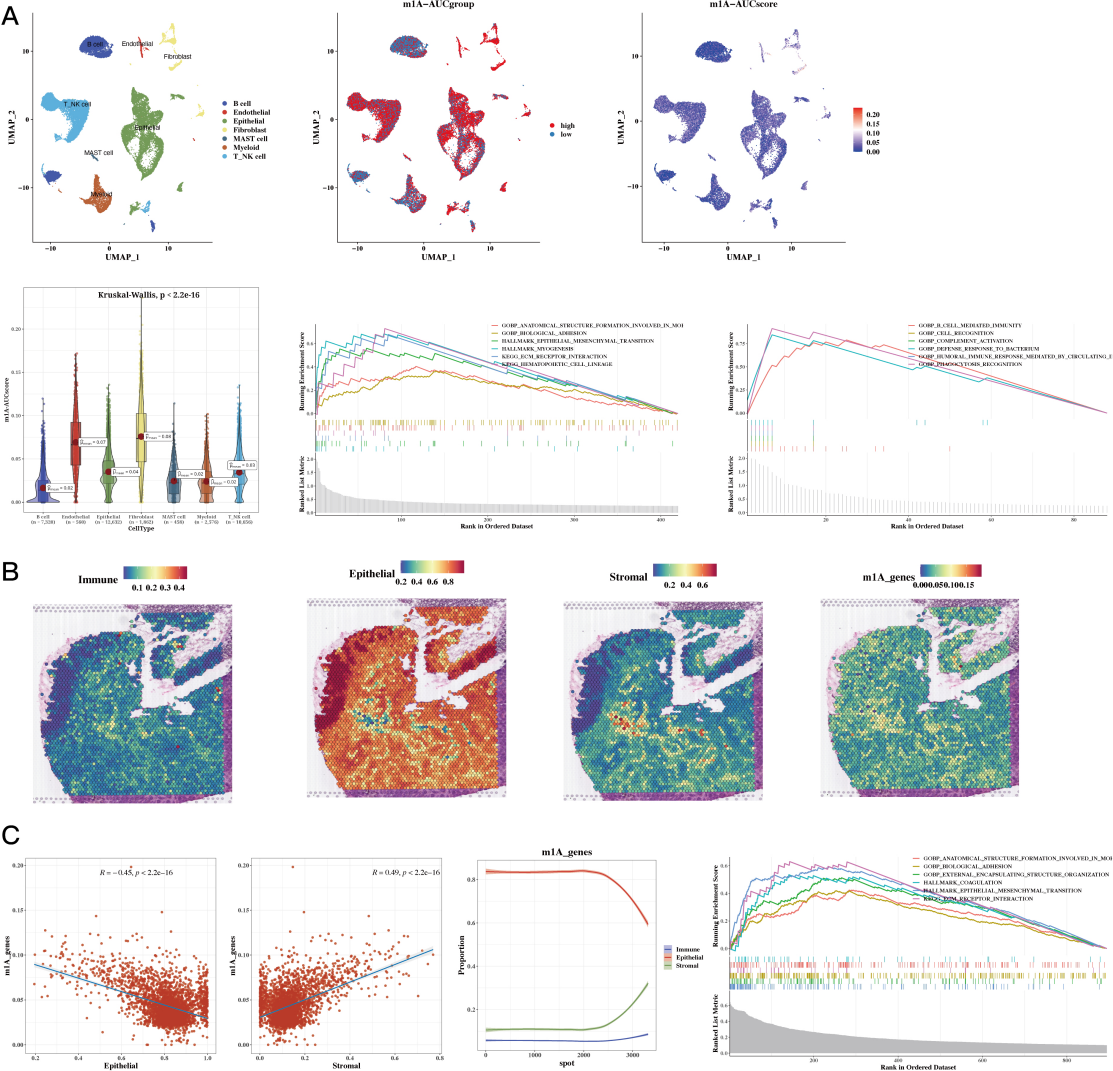
Functional Characterization - Single-Cell and Spatial Transcriptomics Results. **(A)** Dingle-cell analysis showing cell annotations, m1A grouping, and m1A scores (UMAP plots); m1A scores (violin plot); functional enrichment in high and low m1A groups (GSEA plot). **(B)** H&E staining showing immune, epithelial, stromal, and m1A scores in spatial transcriptomic data. **(C)** Epithelial, stromal scores, and m1A scores in spatial transcriptomic data (correlation plots); epithelial, immune, and stromal scores sorted by ascending m1A scores (line plot); functional enrichment in high m1A group (GSEA plot).

The results of cell classification using single-cell data were presented, and the m1A score for each cell was calculated using the AUCell package for the 35 prognostic genes. The results revealed that stromal cells had a higher score. Using the AUCell_exploreThresholds function in the AUCell package, the cells were divided into two groups, and the differential genes of the two groups were calculated. Enrichment analysis was conducted to explore functional differences between these two groups (see **Figure 3A**). The results of our analysis of the spatial transcriptomic sample revealed differences in the distribution of immune, epithelial, and stromal cells; we also calculated the m1A score in the spatial transcriptomic sample. The results showed that, consistently with our single-cell findings, the regions with high m1A scores were mainly in the stromal area (see **Figure 3B**). The results also showed a negative correlation between epithelial cells and m1A score, while immune and stromal cells were positively correlated. Functional enrichment analysis was then performed for the high m1A group (see **Figure 3C**).

### 3.4 Prognostic model construction based on differential genes

**Figure 4:**
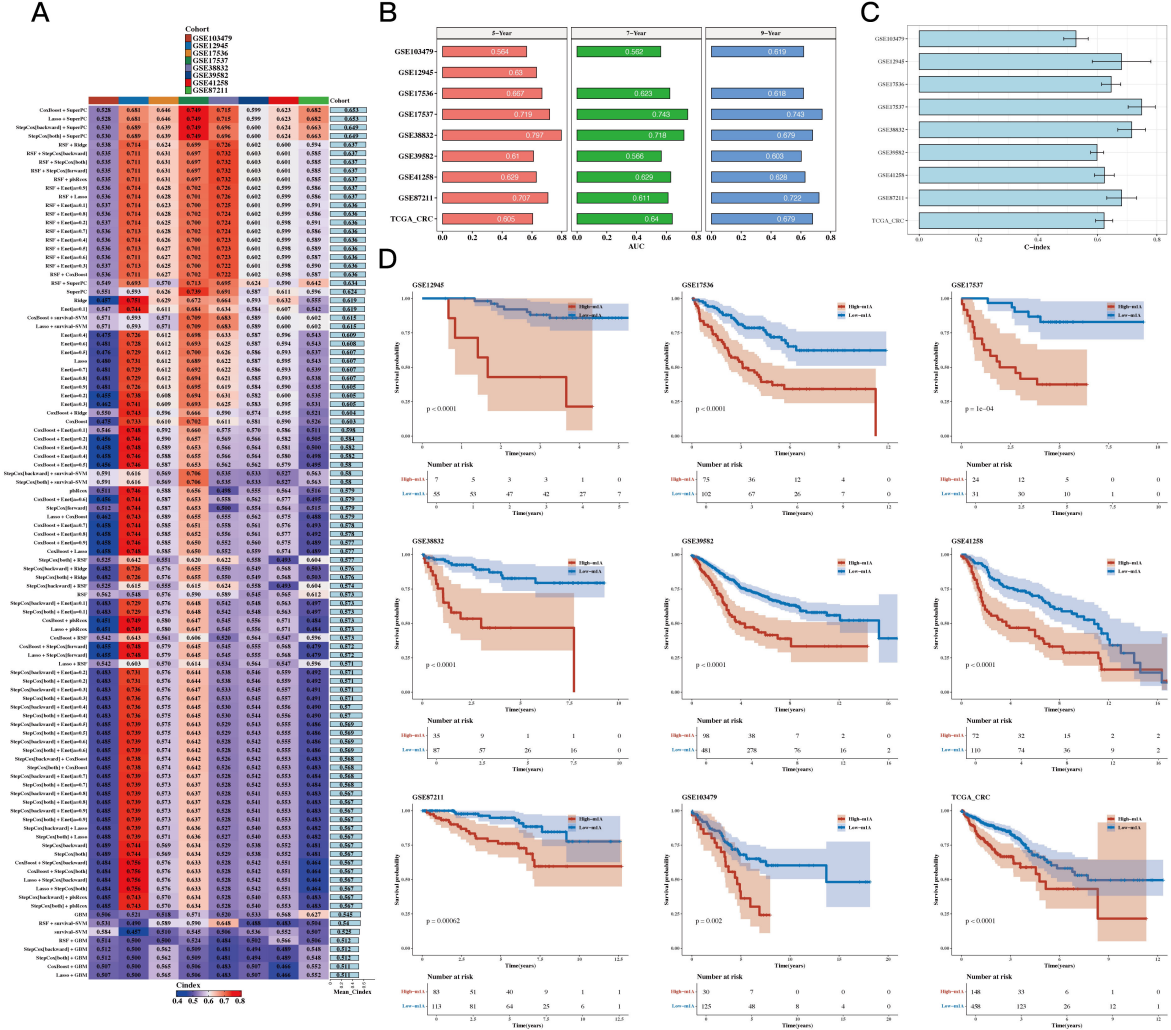
Construction Results of the Prognostic Model Based on Differential Genes. **(A)** C 101 algorithms and eight validation datasets (C-index heatmap). **(B)** Area under the curve (AUC) values at 5, 7, and 9 years for 9 datasets. **(C)** C-index for the optimal model across all datasets (bar plot). **(D)** Prognosis differences between high- and low-risk groups (survival analysis results for 9 datasets).

Using TCGA as the training set and eight GEO datasets as the test sets, we constructed a prognostic model using 12 prognostic genes with 101 different algorithms, using TCGA as the training set and eight GEO datasets as the test sets. The average C-index of the eight test sets was used as the evaluation criterion, and Coxboost+SuperPC was determined as the best model (see **Figure 4A**). We then calculated the 5-, 7-, and 9-year AUC values for the 9 datasets (see **Figure 4B**) and presented a bar chart of the C-index for the optimal model across all datasets (see **Figure 4C**). The survival analysis results for the 9 datasets indicated that the high-risk group had a worse prognosis (see **Figure 4D**).

### 3.5 Comparison of prognostic models

**Figure 5:**
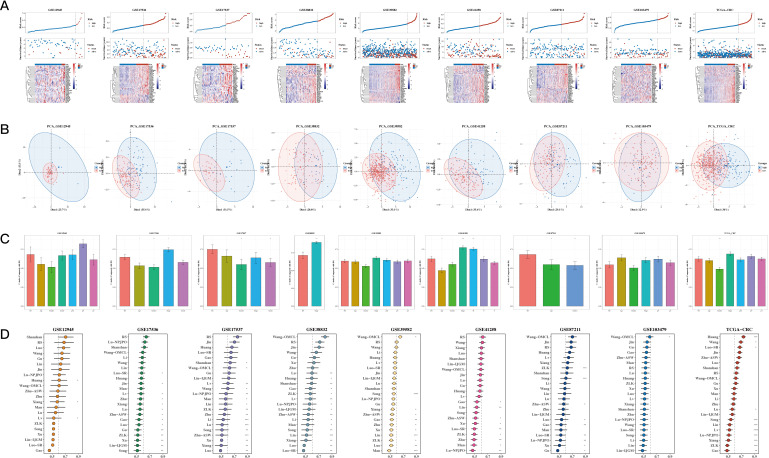
Comparison of Prognostic Models. **(A, B)** Risk plots and principal component analysis (PCA) plots for nine datasets. **(C)** Bar The C-index of risk scores with other clinical indicators (bar plot). A p value < 0.05 was considered statistically significant (* p < 0.05; ** p < 0.01; *** p < 0.001). **(D)** C-index comparison between our prognostic model and 22 other published prognostic models across 9 datasets.

Risk plots and PCA plots for the 9 datasets are shown in **Figures 5A, B**. The risk scores were compared with other clinical indicators, showing that the C-index of the risk score was superior to most clinical indicators (see **Figure 5C**). We also collected 22 recently published prognostic models from the past 1-2 years and compared their C-indices. The results revealed that, although our model was not superior in the TCGA cohort, it generally outperformed most other models in the remaining eight test datasets (see **Figure 5D**).

### 3.6 Establishment of the nomogram model

**Figure 6:**
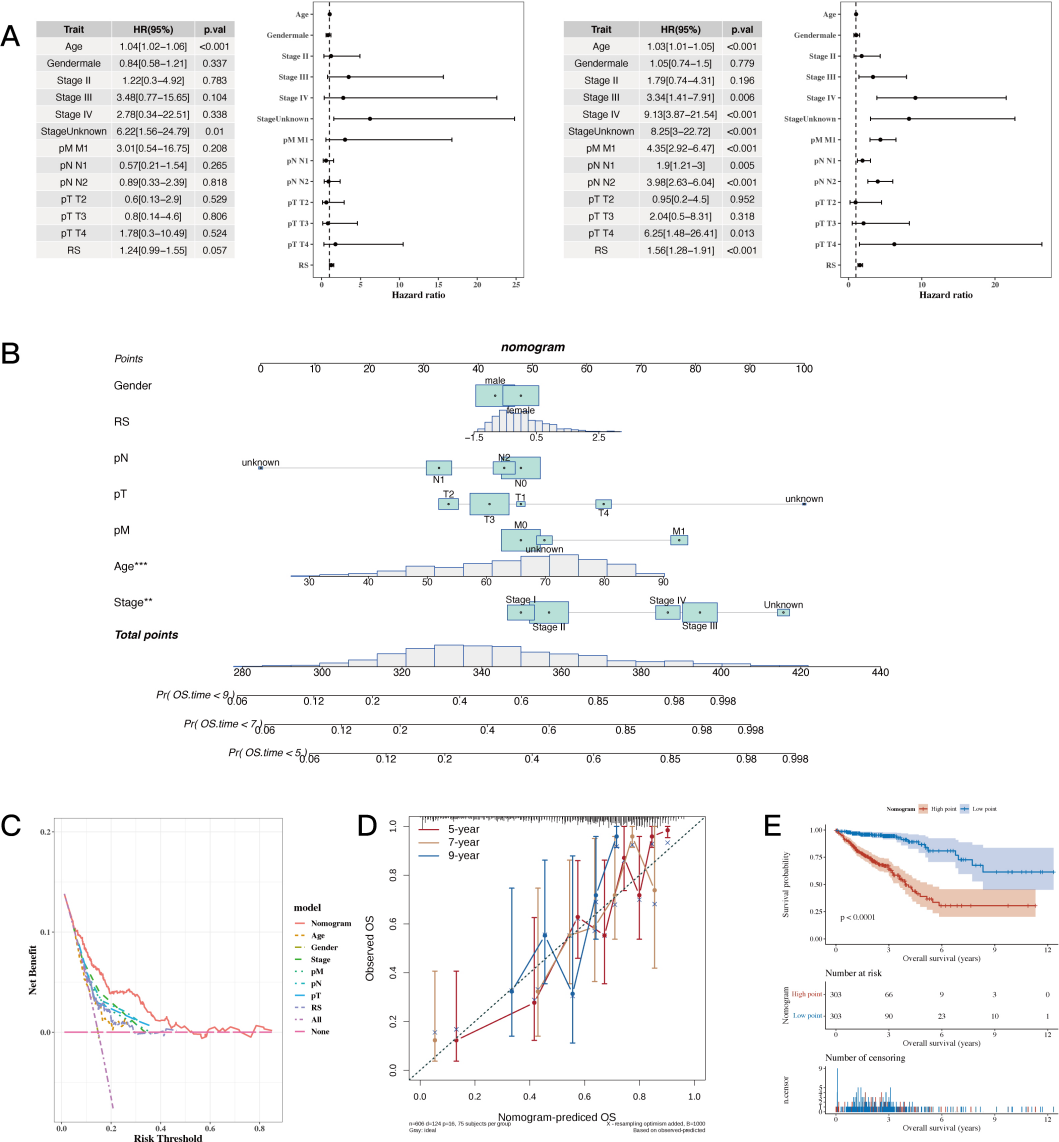
Establishment Results of the Nomogram Model. **(A)** Risk scores and clinical indicators (univariate and multivariate analysis results with forest plot). **(B)** Nomogram plot combining risk scores with clinical indicators for individualized risk prediction. **(C, D)** Decision curve analysis (DCA) plot and calibration curves for 5, 7, and 9 years, respectively. **(E)** Survival analysis plot using nomogram scores to assess prognosis. A p value < 0.05 was considered statistically significant (* p < 0.05; ** p < 0.01; *** p < 0.001).

The results of univariate and multivariate analysis results, along with the forest plot for risk score and clinical indicators, are shown in **Figure 6A**. A nomogram combining risk scores with clinical indicators was constructed (**Figure 6B**). Decision curve analysis (DCA) showed that the nomogram and risk score outperformed other clinical indicators (see **Figure 6C**). Calibration curves for 5, 7, and 9 years are shown in **Figure 6D**. The results of survival analysis using the nomogram score revealed that a higher score was associated with poorer prognosis (see **Figure 6E**).

### 3.7 Tumor immune infiltration analysis and TMB analysis

**Figure 7:**
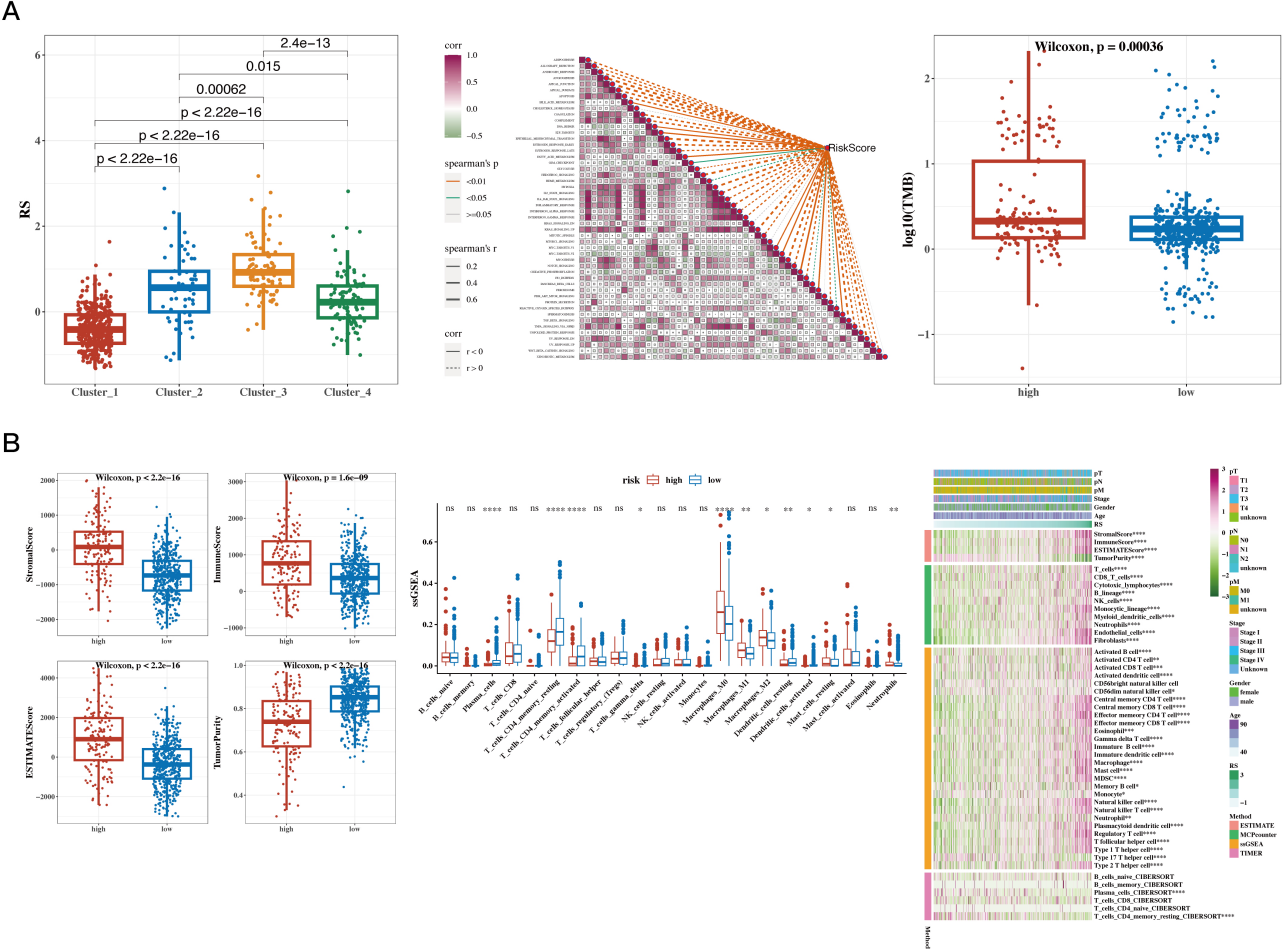
Results of Tumor Immune Infiltration Analysis and TMB Analysis. **(A)** Risk values for the four NMF groups (box plot); correlation between risk scores and 50 hallmark gene sets (heatmap); tumor mutational burden (TMB) between high- and low-risk groups (box plot). **(B)** Differences in immune, stromal, ESTIMATE scores, and tumor purity between high- and low-risk groups (box plot); CIBERSORT analysis comparing immune cell infiltration between the two groups (box plot); infiltration levels estimated by additional immune infiltration algorithms (heatmap). A p value < 0.05 was considered statistically significant (* p < 0.05; ** p < 0.01; *** p < 0.001).

The risk values were significantly different among the four NMF classification groups (see **Figure 7A**). Correlation analysis was performed between the risk scores and 50 hallmark gene sets (see **Figure 7A**). Tumor mutational burden (TMB) was calculated using mutation data, and significant differences were found between risk groups (**Figure 7A**). The differences in immune, stromal, ESTIMATE scores, and tumor purity between the two groups were presented, followed by the use of the CIBERSORT algorithm to show differences in immune cell infiltration. Further analysis using other algorithms such as MCP-counter and TIMER was conducted to estimate immune infiltration levels, and the correlation with risk scores was displayed using a heatmap (see **Figure 7B**).

### 3.8 Immunotherapy analysis and drug sensitivity analysis

**Figure 8:**
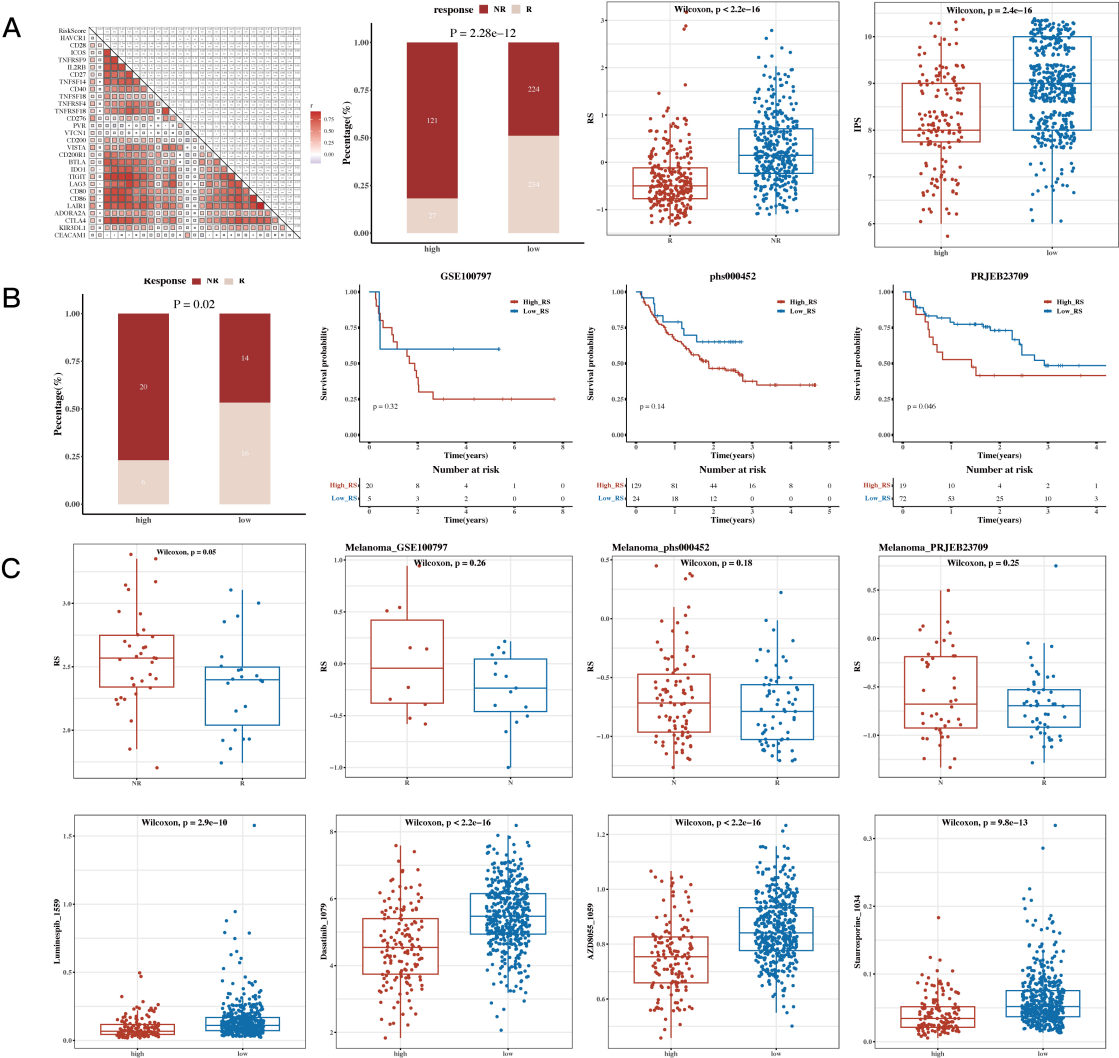
Immunotherapy Analysis and Drug Sensitivity Analysis Results. **(A)** Risk scores and immune checkpoint genes (correlation heatmap); TIDE (Tumor Immune Dysfunction and Exclusion) composition (bar plot); TIDE risk values (box plot); IPS (Immunophenoscore, box plot). **(B)** Survival analysis results and risk scores for immune response groups in immunotherapy cohorts GSE100797 (Melanoma), phs000452 (Melanoma), PRJEB23709 (Melanoma), and GSE35640 (Melanoma). **(C)** Sensitivity of drugs Staurosporine_1034, Luminespib_1559, Dasatinib_1079, and AZD8055_1059 between high- and low-risk groups (box plots).

The results of correlation analysis conducted between immune scores and commonly used immune checkpoint genes revealed that most of the genes were positively correlated. The TIDE algorithm, which was used to predict immune response in the TCGA dataset, showed significant differences between response and non-response groups, with the non-response group having higher risk scores. The IPS results were also incorporated, showing that the low-risk group had higher IPS scores (see **Figure 8A**). This heatmap displays the distribution of IPS scores across multiple cohorts, with the color gradient ranging from blue to red to represent lower to higher IPS scores, respectively. Each row corresponds to an immune-related pathway or cell type, while the columns represent the cohorts analyzed. The visualization highlights distinct patterns of immune activity, supporting the conclusion that the low-risk group exhibits a more favorable immune profile. The results of survival analysis and risk scores for immune response groups were presented for the GSE100797 (Melanoma), phs000452 (Melanoma), PRJEB23709 (Melanoma), and GSE35640 (Melanoma) datasets (see **Figure 8B**). The results of drug sensitivity analysis showed that Staurosporine_1034, Luminespib_1559, Dasatinib_1079, and AZD8055_1059 were sensitive to the high-risk group, suggesting their suitability for treatment of patients in this group (see **Figure 8C**).

### 3.9 Differences in cell communication of high- and low-risk cells at the single-cell level

**Figure 9:**
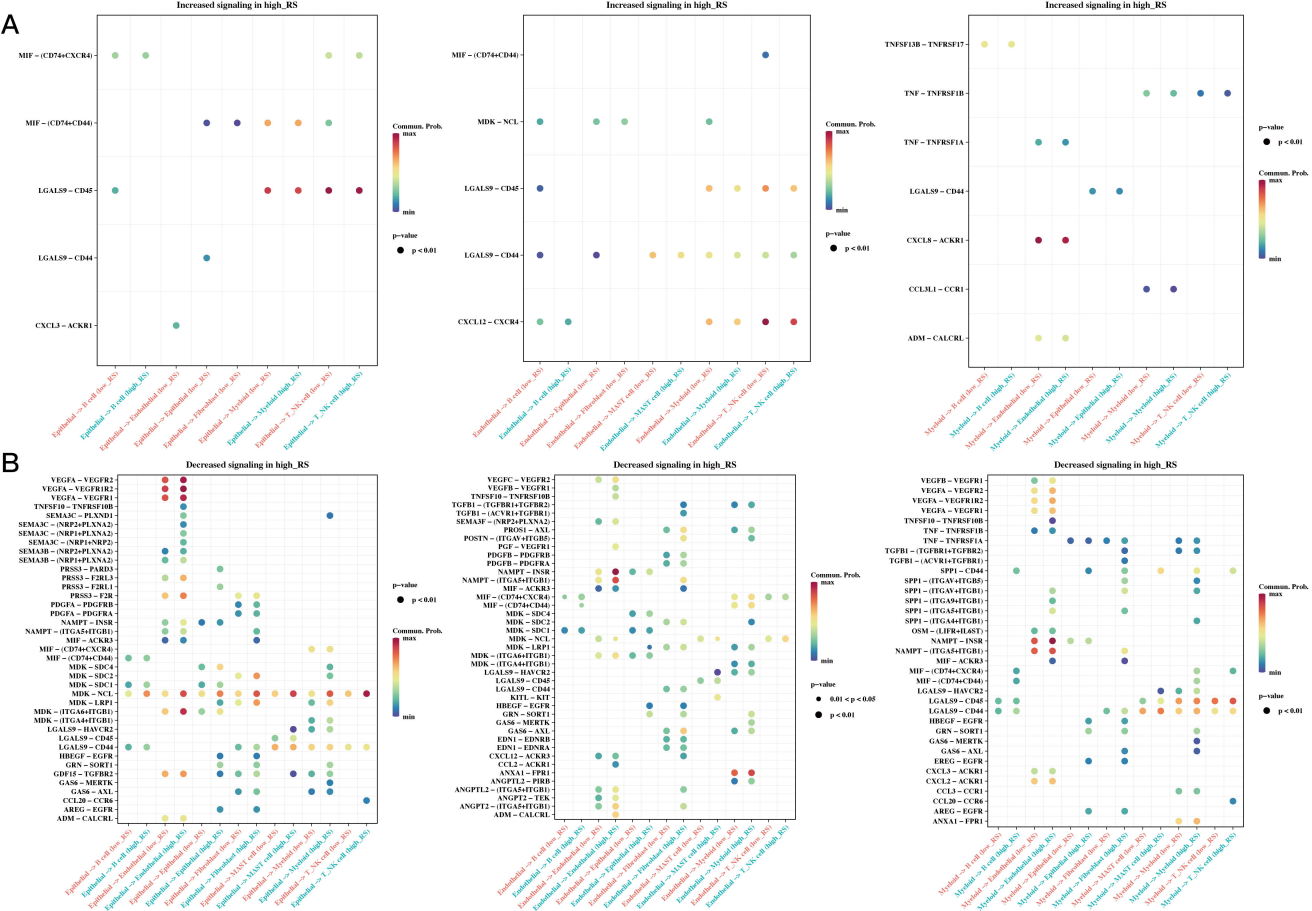
Differences in Cell Communication of High- and Low-Risk Cells at the Single-Cell Level. **(A)** Communication differences in epithelial, endothelial, and myeloid cells between high- and low-risk groups, focusing on interactions increased in the high-risk group (bubble plot). **(B)** Communication differences in epithelial, endothelial, and myeloid cells between high- and low-risk groups, focusing on interactions decreased in the high-risk group (bubble plot).

We calculated risk scores for each cell in the single-cell dataset using the risk model, grouped the cells based on the median value, and then conducted CellChat cell communication analysis to compare differences between the two groups. **Figure 9** shows differences in communication among epithelial, endothelial, and myeloid cells between the high- and low-risk groups.

### 3.10 SLC12A2 exhibits oncogenic properties in colorectal cancer cells

**Figure 10:**
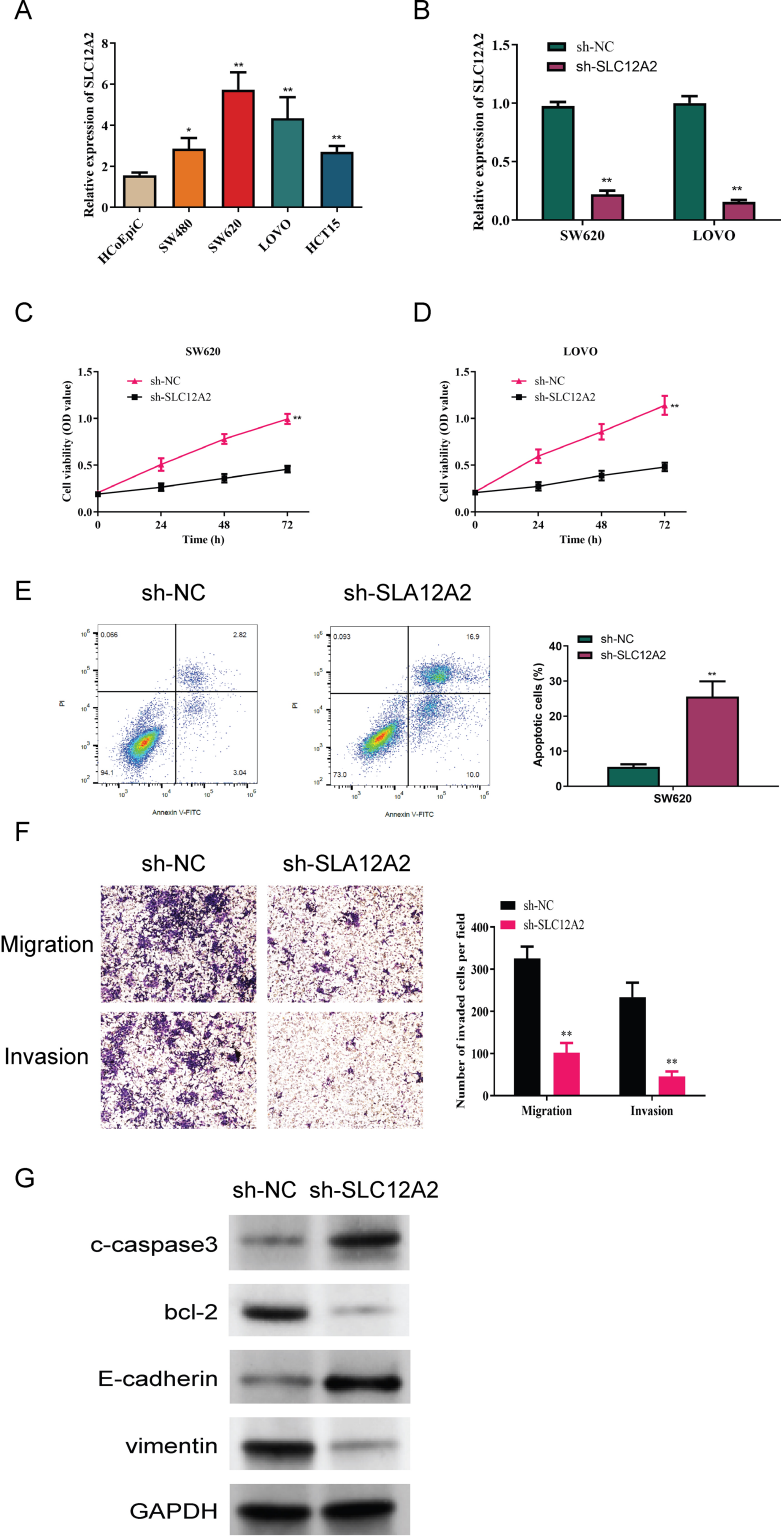
Experimental data analysis. **(A)** Relative expression of SLC12A2 in various cell lines, indicating low expression in HCoEpiC cells and high expression in SW480, SW620, LOVO, and HCT15 cells (*p* < 0.01). **(B)** Relative expression of SLC12A2 across different experimental groups, with efficient knockdown observed in two cell lines. **(C, D)** Knockdown of SLC12A2 significantly reduces the proliferation capacity of cancer cells (CCK-8 assay results). **(E)** Knockdown of SLC12A2 significantly increases the apoptotic rate of cancer cells (flow cytometry analysis). **(F)** Knockdown of SLC12A2 markedly inhibits the invasion and migration of cancer cells (transwell assays). **(G)** Thesh-SLC12A2 group exhibits elevated expression of E-cadherin and cleaved caspase-3, along with reduced expression of Bcl-2 and vimentin (Western blot analysis).

We first analyzed SLC12A2 expression in the normal colonic epithelial cell line HCoEpiC, as well as in the following four colorectal cancer cell lines: SW480, SW620, LOVO, and HCT15. This analysis aimed to compare the expression levels of SLC12A2 across these different cell lines to better understand its potential role in colorectal cancer. A marked upregulation of SLC12A2 in colorectal cancer cells was observed (*p* < 0.05, **Figure 10A**). the results of RT-qPCR confirmed effective knockdown of SLC12A2, with SW620 sh-SLC12A2 and LOVO sh-SLC12A2 cell lines exhibiting significantly reduced SLC12A2 expression (*p* < 0.01, **Figure 10B**). The findings of the CCK-8 assay demonstrated that silencing SLC12A2 led to a marked decrease in the viability of SW620 and LOVO colorectal cancer cells. This finding indicates that SLC12A2 is essential for enhancing cell proliferation in these cancer cell lines, highlighting its potential role as an oncogene in colorectal cancer (*p* < 0.01, **Figures 10C, D**). Furthermore, the results of flow cytometry revealed a significant increase in apoptosis following SLC12A2 knockdown (*p* < 0.01, **Figure 10E**). Transwell assay results showed that SLC12A2 knockdown significantly impaired the invasive capacities and the migratory of SW620 and LOVO cells, suggesting that SLC12A2 is crucial for the metastatic potential of colorectal cancer cells (*p* < 0.01, **Figure 10F**). The Western blot analysis showed that the sh-NC group expressed Bcl-2 and vimentin at normal levels, whereas the sh-SLC12A2 group exhibited increased expression of E-cadherin and c-caspase3. However, the levels of Bcl-2 and vimentin were found to be reduced. suggesting that knockdown of SLC12A2 led to significant alterations in the expression of molecules associated with migration, invasion, and apoptosis (see **Figure 10G**). Based on the results, it can be concluded that SLC12A2 plays an oncogenic role in colorectal cancer cells, promoting their proliferation, invasion, and migration.

## 4 Discussion

In this study, we conducted an in-depth analysis of m1A-related genes in colorectal cancer (CRC). The specific focus was on evaluating their prognostic potential, understanding their relationship with tumor molecular subtypes, and exploring their role in shaping the tumor microenvironment (TME). The results provide new insights into the significance of m1A modifications in CRC, highlighting their potential utility in prognosis prediction and as therapeutic targets. In this discussion, we compare our results with the previously reported findings, review implications of our findings, and outline the strengths and limitations of the study.

Colorectal cancer, one of the leading causes of cancer mortality worldwide, is characterized by a significant heterogeneity in its clinical outcomes. The characterization of m1A-related genes has emerged as a promising avenue for understanding tumor biology and identifying novel biomarkers for CRC. RNA modifications, particularly N1-methyladenosine (m1A), have recently gained considerable research attention for their role in post-transcriptional regulation of gene expression; however, the role of RNA modifications in CRC has not been extensively studied. Our results demonstrate that consistently with recent studies that demonstrated the clinical relevance of other RNA modifications, such as N6-methyladenosine (m6A), in various cancers (31), m1A-related genes can serve as valuable biomarkers for prognosis in CRC.

Using performing univariate Cox analysis across multiple datasets, including TCGA and GEO, we identified 43 m1A-related genes significantly associated with CRC prognosis. These genes were subsequently used for molecular subtyping, revealing four distinct subtypes with significantly different survival outcomes. This stratification allowed us to identify groups with notably better or worse prognoses, such as C1 and C3, respectively. The identification of distinct molecular subtypes is in line with previous research on CRC that demonstrated extensive genetic and molecular heterogeneity (9). Previous studies that identified consensus molecular subtypes (CMS) of CRC showed that distinct subgroups of CRC patients have different outcomes and responses to treatment (9). Aligning with this concept, our molecular subtyping, based on m1A-related genes, provides additional insights into the impact of epigenetic modifications on CRC prognosis.

One of the major findings of our study was the differential correlation between m1A-related gene expression and immune and stromal scores within the TME. Specifically, stromal cells were found to exhibit higher m1A scores, while the regions with elevated m1A scores were predominantly located in stromal areas. This suggests that m1A-related genes may play a significant role in modulating stromal components and shaping the TME in CRC. The TME plays a critical role in cancer progression, immune evasion, and response to therapy, and recent studies demonstrated that alterations in RNA modifications can significantly influence the TME (32). For example, previous research revealed that m6A RNA modifications are involved in immune cell recruitment and immune evasion in various cancers (32–34). Similarly, our results suggest that m1A-related genes may have a regulatory role in TME dynamics in CRC, affecting not only stromal composition, but also immune cell infiltration.

Furthermore, using immune infiltration analysis, we found that m1A-related gene expression was positively correlated with immune checkpoint genes, indicating potential implications for immunotherapy. The TIDE algorithm showed that low-risk groups, as defined by our prognostic model, exhibited a better predicted response to immune checkpoint inhibitors (ICIs) compared to high-risk groups. These findings are consistent with recent studies indicating that RNA modifications can affect immune cell infiltration and modulate responses to immunotherapy (35). Available evidence suggests that patients with high levels of m6A modifications exhibited improved responses to ICIs in melanoma (35). Similarly our results show that m1A-related genes may serve as predictive biomarkers for immunotherapy in CRC, suggesting their role in the immune modulation of CRC tumors.

In addition to bulk RNA-sequencing analyses, we also employed single-cell RNA sequencing (scRNA-seq) to investigate heterogeneity of m1A-related gene expression on the single-cell level. scRNA-seq allowed us to identify distinct cell populations in the TME that exhibited different m1A scores, providing insights into the cell-specific functions of m1A modifications in CRC. For instance, epithelial cells had lower m1A scores as compared to stromal and immune cells, suggesting differential roles of m1A modifications across different cell types within the tumor. This finding is consistent with previous research showing that the distribution and function of RNA modifications can significantly vary among different cell types, influencing their phenotype and behavior in the tumor context (33). Given that the effects of such modifications can differ depending on the cell type and its role in the TME, oresults underscore the importance of considering cellular heterogeneity when studying epigenetic modifications in cancer.

We also explored cell-cell communication using the CellChat algorithm, which revealed differences in communication patterns between high- and low-risk cells, particularly among epithelial, endothelial, and myeloid cells. These results suggest that m1A-related genes may affectcell-cell communication within the TME, thereby contributing to CRC progression and therapeutic resistance. Altered intercellular signaling is a hallmark of cancer, and the TME plays a central role in facilitating tumor-promoting interactions between cancer cells and their microenvironment. Our findings highlight that m1A modifications may be key regulators of intercellular communication in CRC, affecting signaling pathways that promote tumor growth and immune evasion (36).

Another major contribution of this study is the construction and validation of a prognostic model based on m1A-related genes. Using 101 different algorithms, we identified the optimal model, using TCGA as the training set and several GEO datasets as test sets. The final model, Coxboost+SuperPC, demonstrated robust prognostic performance across multiple datasets, outperforming other recently published models in most cases. The model’s superior predictive ability, as evidenced by its higher C-index and area under the curve (AUC) values, highlights its potential utility for clinical applications in CRC. Developing accurate prognostic models is essential for guiding treatment decisions, particularly in heterogeneous diseases like CRC. In this context, the proposed model, which integrates m1A-related gene expression with clinical indicators in a nomogram, provides a practical tool for individualized risk assessment and personalized treatment planning (37).

The implications of our findings extend beyond prognosis. Specifically, the positive correlation between m1A-related genes and immune checkpoints suggests that these genes could serve as potential biomarkers for selecting patients who are more likely to respond to ICIs. Considering the increasing use of immunotherapy in CRC, especially for patients with microsatellite instability-high (MSI-H) tumors, it is crucial to identify biomarkers that predict response to these therapies. While MSI status is currently used as a biomarker for immunotherapy in CRC, our findings suggest that m1A-related gene expression could complement existing markers and enhance patient selection for immunotherapy (38).

We also found that the expression levels of SLC12A2 were significantly higher in colorectal cancer cell lines than in normal cell counterparts, suggesting its potential role in cancer development. This finding may highlight SLC12A2 as a candidate biomarker or therapeutic target in colorectal cancer. Furthermore, SLC12A2 knockdown significantly reduced the viability of colorectal cancer cells, increased apoptosis, and diminished both migratory and invasive capabilities. Our experimental results demonstrated that SLC12A2 promotes CRC cell proliferation, migration, and invasion while inhibiting apoptosis. Mechanistically, knockdown of SLC12A2 increased cleaved caspase-3 and E-cadherin levels while reducing Bcl-2 and vimentin, indicating its role in apoptotic and epithelial-mesenchymal transition (EMT)-related pathways. Based on this evidence, we hypothesize that m1A methylation may regulate SLC12A2 expression by modifying mRNA stability or translation efficiency. This hypothesis will be validated in our future experiments, such as MeRIP-qPCR and mutagenesis. In addition, the results of immune and stromal analyses revealed that m1A-related gene expression positively correlates with immune checkpoint genes and immune cell infiltration, suggesting their role in immune evasion and response to immunotherapy. Single-cell and spatial transcriptomics further demonstrated cell type-specific and spatially organized expression patterns of m1A-related genes, linking them to stromal and immune cell regulation within the tumor microenvironment (TME). Taken together, these findings provide a foundation for understanding the mechanistic roles of m1A-related genes in CRC and outline directions for future research.

The present study has several limitations. First, our analysis was based on publicly available datasets, and the results may have been affected by the quality and heterogeneity of these datasets. Although we validated our findings across multiple datasets, prospective validation using independent cohorts, preferably from different populations, would be necessary to confirm the robustness and generalizability of our results. Second, in this study, we did not yet experimentally validate the functional roles of the identified m1A-related genes in CRC. While we provided evidence for their association with prognosis and immune infiltration, further mechanistic studies would be needed to elucidate how these genes regulate CRC progression and response to therapy. *In vitro* and *in vivo* studies could help confirm the causal relationships between m1A modifications and the observed effects on tumor behavior and the TME (38).

Another limitation is the lack of exploration into the potential interplay between m1A and other RNA modifications, such as m6A, m5C, and pseudouridine. These RNA modifications frequently coexist and can interact to modulate gene expression in a coordinated manner. To better understand the overall epitranscriptomic regulation in CRC, in future research, it would be meaningful to investigate the combinatorial effects of different RNA modifications. In addition, while our single-cell analyses provided valuable insights into the heterogeneity of m1A-related gene expression, integrating spatial transcriptomics with single-cell data could provide further information on the spatial organization of these modifications within the TME.

In conclusion, our study provides a comprehensive analysis of m1A-related genes in CRC, highlighting their role in shaping tumor heterogeneity, influencing immune infiltration, and serving as valuable prognostic biomarkers. By integrating multi-omics data, characterizing molecular subtypes, along with evaluating the prognostic value of m1A-related genes, we identified novel markers that could inform precision oncology for CRC patients. The construction of a robust prognostic model and its validation across multiple datasets further supports the clinical relevance of these genes providing a novel tool for personalized risk assessment and treatment planning. Our findings suggest that m1A-related genes could serve as potential targets for therapeutic intervention, particularly in the context of immunotherapy. Future research should focus on experimental validation of these findings and further exploration of the mechanisms underlying the role of m1A modifications in CRC.

## Data Availability

The original contributions presented in the study are included in the article/supplementary material. Further inquiries can be directed to the corresponding author.
